# The morphoanatomy of *Serjania erecta* Radlk (Sapindaceae) provides evidence of biotrophic interactions by endophytic fungi within leaves

**DOI:** 10.7717/peerj.15980

**Published:** 2023-09-15

**Authors:** Samylla Tássia Ferreira de Freitas, Giselle Faria, Fabiano Guimarães Silva, Marco Aurélio Batista, Damiana Souza Santos Augusto, Fábio Henrique Dyszy, Luciana Cristina Vitorino

**Affiliations:** 1 Graduate Program in Agricultural Sciences, Instituto Federal de Educação, Ciência e Tecnologia Goiano (IF Goiano, Campus Rio Verde), Rio Verde, Brazil; 2 Graduate Program in Natural Resources of the Cerrado, Universidade Estadual de Goiás, Anápolis, Brazil; 3 Graduate Program in Biodiversity and Conservation, Instituto Federal de Educação, Ciência e Tecnologia Goiano (IF Goiano, Campus Rio Verde), Rio Verde, Brazil

**Keywords:** Five-leaf liana, Bioactive compounds, Leaf teeth, Biotrophic fungi, Trichomes

## Abstract

**Background:**

The leaves of *Serjania erecta* Radlk (Sapindaceae) are renowned in ethnobotany for their medicinal properties and are significant as a medicinal resource for traditional Brazilian communities. As necrotic spots are common on these leaves, indicating interaction with phytopathogenic fungi, it was hypothesized that biotrophic fungal species colonize the leaf tissues of *S. erecta*.

**Methods:**

To test this hypothesis, we employed standard techniques in plant anatomy, which enabled us to investigate the interaction of fungal structures with plant tissues and describe the morphoanatomical and histochemical characteristics of the epidermis and limbus of *S. erecta*.

**Results:**

The anatomical analysis showed the existence of leaf teeth on the leaf tips. Additionally, hyphae, conidiospores, and spores of *Bipolaris/Curvularia* species were detected on the adaxial epidermis. Moreover, melanized microsclerotia were found in glandular areas of the leaf teeth and the phloem, providing evidence of biotrophic behavior. The hypothesis that biotrophic phytopathogenic fungi interact with *S. erecta* leaf tissues was confirmed, despite the presence of many bioactive compounds (such as flavonoids, alkaloids, and essential oils), as evidenced by histochemical analyses. The presence of tector, glandular, and scabiform trichomes on the leaf teeth and epidermis was also revealed. This study presents, for the first time, the synthesis of essential oils and alkaloids in the leaves of *S. erecta*. Additionally, it investigates previously unexplained aspects of the anatomy and histochemistry of the species, as well as its interaction with resident microorganisms. Therefore, it is recommended that future research focus on extracting and characterizing the oils and alkaloids of *S. erecta*, as well as exploring other aspects related to its microbiome and its relationship.

## Introduction

Sapindaceae is the largest and most important family of the order Sapindales (APG IV, Angiosperm Phylogeny Group IV), with about 140 genera and 1,900 species mainly distributed in tropical regions (Acevedo-Rodríguez et al., 2011). In Brazil, the family is represented by 32 genera and 436 species, of which 191 are endemic (Flora do Brasil, 2020). This family comprises many species of economic and medicinal importance (Heywood et al., 2007). Among them *Serjania erecta* Radlk, commonly known as Cipó de cinco folhas. *S. erecta* is characterized by erect subshrubs with flexuous growth. Its leaves are compound, with leaflets up to 15 cm long and up to 10 cm wide. Additionally, it has inflorescences in solitary thyrsus with white flowers. The fruits are schizocarpic, slightly corded, and up to 3.5 cm long, with subglobose and slightly arilate seeds (Guarim Neto & Macedo, 2009). It is a native plant that grows throughout the Brazilian cerrado and has been reported for its various ethnobotanical applications, including its use in teas and extracts to treat ulcers and inflammatory infections (Rodríguez & Pinto, 2014; Guarim Neto, Santana & Silva, 2000). The populations living along the riverside of the Paraná and Cuiabá River basins use tea made from plant leaves to alleviate flu symptoms (Reis & Bellini, 2007). Additionally, there are reports that indigenous communities in Mato Grosso State use the roots of the plant to manage hypertension (Guarim Neto & Macedo, 2009).

The biological effects attributed to *S. erecta* in traditional and indigenous medicine may be related to the bioactive compounds found in this species. Qualitative analyses have already identified the presence of saponins, flavonoids, triterpenoids, steroids, tannins, and catechins in the hydroalcoholic extract of the leaves (Gomig et al., 2008; Ventura et al., 2021). Similarly, analyses of the crude extract using nuclear magnetic resonance showed the presence of flavonoids such as camferol, epicatechins, apigenins, vitexin, isovitexin, and quercetin (Guimarães et al., 2015; Cardoso et al., 2013).

Research demonstrates that the interaction between *S. erecta* leaf extract, animals, and microbial cells produces various effects. The hydroalcoholic extract showed topical anti-inflammatory activity in mice (Gomig et al., 2008). Additionally, the chloroform extract exhibited gastroprotective action and reduced the ulcerative process (Arruda et al., 2009). Memory disorders appear to be mitigated by the use of the crude extract of the plant, which has a synergistic effect with pharmacological drugs (Broggini et al., 2010).

Other trials have shown nematicidal effects against *Pratylenchus zeae* and *P. jaehni* (Slomp et al., 2009), as well as antimicrobial effects against key human pathogens, including *Mycobacterium tuberculosis*, *Staphylococcus aureus*, *Pseudomonas aeruginosa*, *Salmonella setubal*, *Candida albicans*, *Saccharomyces cerevisiae*, and *Escherichia coli* (Cardoso et al., 2013). Metabolites present in *S. erecta* can also affect insects, as it directly interferes with the growth, metabolism, and development of caterpillars (De Freitas et al., 2022).

Despite the considerable phytochemical potential of *S. erecta*, extensive studies on this plant are scarce. This species has not yet been evaluated for its anatomy and histochemistry, although studies describe its biotechnological potential and promote its conservation. According to Guarim Neto & Macedo (2009), the conservation of this species deserves more attention, considering the serious and irreversible losses caused to the Cerrado domain.

Furthermore, there needs to be more information regarding the morphoanatomical and histochemical aspects of the plant and the microbiota residing in the leaves of *S. erecta*. Given that it is a medicinal plant, any epiphytes or endophytes present must exhibit traits of resistance or insensitivity to the secondary metabolites found in its tissues.

During field observations, it was discovered that *S. erecta* leaves display necrotic spots even on healthy plants, indicating interaction with phytopathogenic fungal species that release toxins and cause local tissue damage (Salvatore & Andolfi, 2021; Doehlemann et al., 2017; Horbach et al., 2011). However, many phytopathogenic species can establish themselves as endophytes and promote plant growth (Abdel-Motaal et al., 2020; Toghueo & Boyom, 2020; Javed et al., 2019). Research has shown that certain necrotrophic pathogens can transition to hemibiotrophy or biotrophy, where they feed on living tissue without causing the death of the host plant.

Biotrophic phytopathogenic fungi were hypothesized to interact with the leaf tissues of *S. erecta*. To test this hypothesis, we employed standard analytical techniques in plant anatomy, which enabled us to investigate not only the interaction of fungal structures with plant tissues but also uncover aspects of the plant’s anatomy and histochemistry not previously elucidated. This study aims to enhance our understanding of the biodiversity of the Cerrado by reducing the information gap regarding the *S. erecta* species. Specifically, we investigated the leaf tissue organization and its interaction with microorganisms that form part of its microbiome.

## Materials & Methods

### Plant material collection

Five *S. erecta* individuals were sampled at Fazenda Fontes do Saber, Rio Verde/GO (−17.783262°S, −50.967928°W), which is characterized by a transitional physiognomy between “cerrado sensu stricto” and “cerradão” vegetation. Field collections were allowed

by the Post-Graduate Direction of the Instituto Federal Goiano Campus Rio Verde (project number: CMRV DPPG 011/2021). The specimens were intended for morphoanatomical characterization analyses, fungal colonization of leaves, histochemical tests using light microscopy, and electron microscopy analyses. Adult specimens with a shrubby habit were collected. An exsiccate was deposited at the herbarium of the Instituto Federal Goiano, Rio Verde campus, for identification confirmation, and it was assigned the catalog number 545.

### Morphoanatomical characterization and evaluation of leaf colonization by fungi

Leaf samples measuring 3 mm^2^ were collected from the central region of the adult leaves of all five individuals of the species. Initially, the samples were fixed in FAA_70_ solution (formalin, acetic acid, and ethyl alcohol) for 24 h. Following this period, the plant material was stored in 70% alcohol and dehydrated using an increasing series of ethyl alcohol. It was then pre-infiltrated and infiltrated with historesin, following the manufacturer’s instructions (Leica, Wetzlar, Germany).

Afterward, the *S. erecta* leaf samples were cut into 5 µm-thick cross-sections using a rotating microtome. The sections were then stained with toluidine blue-polychrome stain (0.05% 0.1 M phosphate buffer, pH 6.8), following the method detailed by O’Brien, Feder & McCully (1964). Images were captured using a microscope with the bright field option. Subsequently, morphoanatomical observations were made on the epidermis of both the adaxial and abaxial faces, as well as the mesophyll. Observations were also made using ultraviolet (UV) light to observe autofluorescent structures.

The leaf diaphanization technique utilized in this study adhered to the protocol outlined by Shobe & Lersten (1967) and Fonsêca, Proença & Gonçalves (2007). Briefly, the fully developed leaves were divided into four sections: apical, middle, margin, and basal thirds. Impressions were taken from the epidermis of both the adaxial and abaxial faces using fresh material and instant adhesive (Segatto et al., 2004); these impressions were then used to prepare semi-permanent slides.

The analyses were conducted at the Plant Anatomy Laboratory of the Instituto Federal Goiano, Campus Rio Verde. The material was photographed using an Olympus microscope (BX61; Olympus, Tokyo, Japan) with a DP-72 camera. Next, the images were recorded using the low gamma option and the highest image contrast. These images were utilized to characterize the morphology and anatomy of *S. erecta* leaf tissues and to evaluate the presence of hyphae and fungal structures colonizing the tissues.

### Scanning electron microscopy

Leaf fragments of approximately 3 mm^2^ were fixed in formalin, acetic acid, and 70% ethyl alcohol and then subjected to a critical point drying process. After compositional analysis, they were coated with gold as a conductive element for image acquisition.

The images were captured using a Jeol JSM7100F field emission scanning electron microscope (SEM-FEG) with a 5 keV electron acceleration voltage in the secondary electron detection (SED) mode. The data was utilized to assess the interaction between hyphae, fungal structures, and *S. erecta* leaf tissues.

### Histochemical tests

To conduct the histochemical tests, serial sections of *S. erecta* leaf samples were first fixed in FAA_70_ solution and then embedded in historesin. These sections were subjected to the following treatments: potassium dichromate (original method: Gabe, 1968) and toluidine blue staining (original method: O’Brien, Feder & McCully, 1964) for identification of phenolic compounds; NADI for essential oils and oleoresins (original method: (David & Carde, 1964); and Schiff’s reagent/periodic acid (PAS) for polysaccharide identification (original method: O’Brien & McCully, 1981). The following tests were performed using fresh material: hydrochloric vanillin for tannin identification (original method: Mace & Howell, 1974); aluminum chloride and fluorescence under UV light for flavonoid identification; and Wagner’s reagent for alkaloid reaction (original method: Furr & Mahlberg, 1981).

## Results

### Leaf morphological characterization

**Figure 1:**
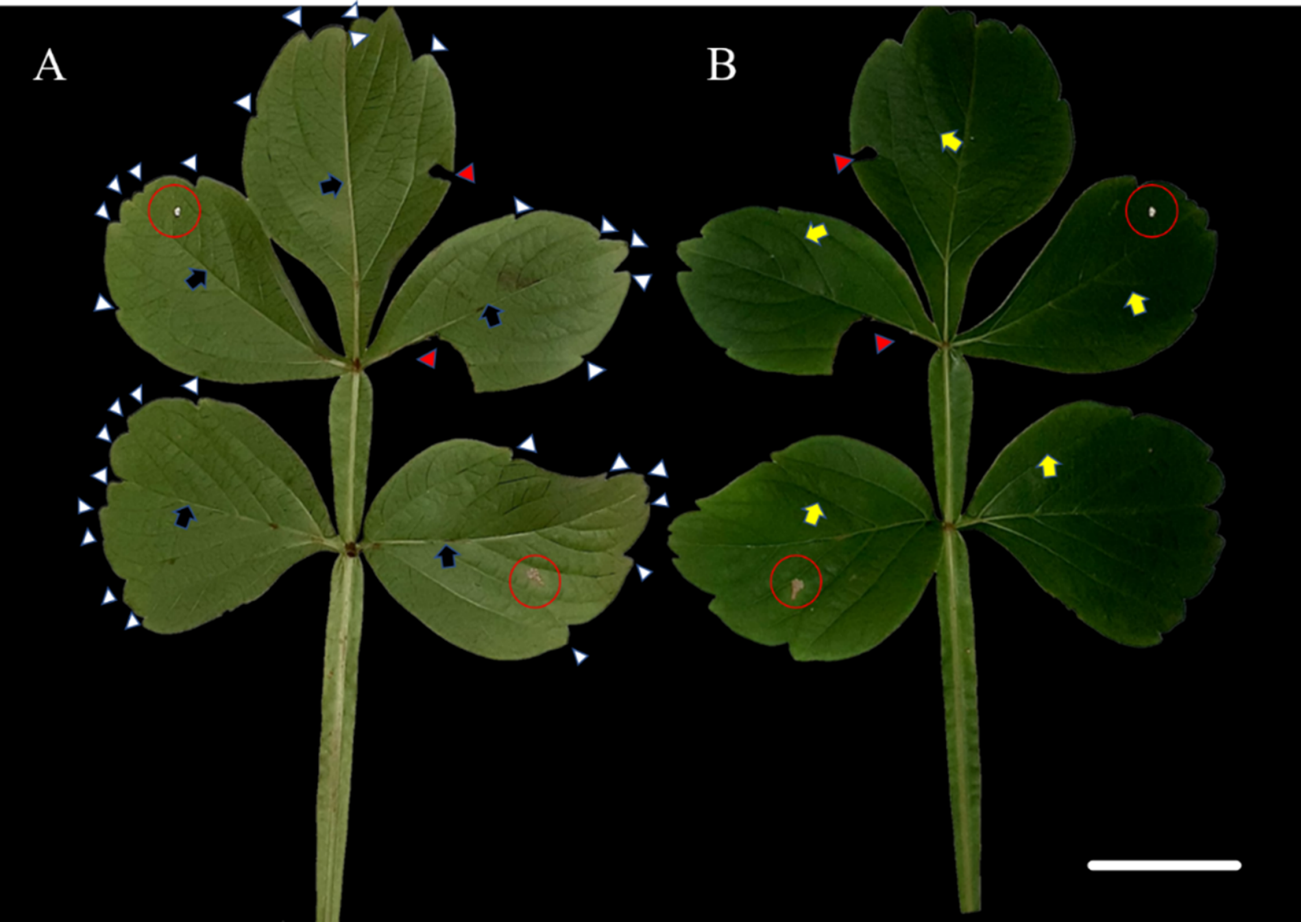
Leaf morphology of *Serjania erecta* Radlk (Sapindaceae). (A) The abaxial surface and (B) the adaxial surface. Red arrows indicate herbivore attacks, while red circles indicate necrotic spots. Blue arrows point to the primary veins; yellow arrows indicate the secondary veins; and white arrows point to the leaf teeth. 5 cm scale bar.

The species *S. erecta* has a compound leaf consisting of five leaflets. Light-colored leaf teeth were present in the apical region of the leaflets (Fig. 1A). Spots of leaf necrosis are a common indication of the interaction between phytopathogenic fungal species and leaf tissues (Figs. 1A–1B). However, the leaf may still retain visible signs of good health.

### Interaction with fungi

**Figure 2:**
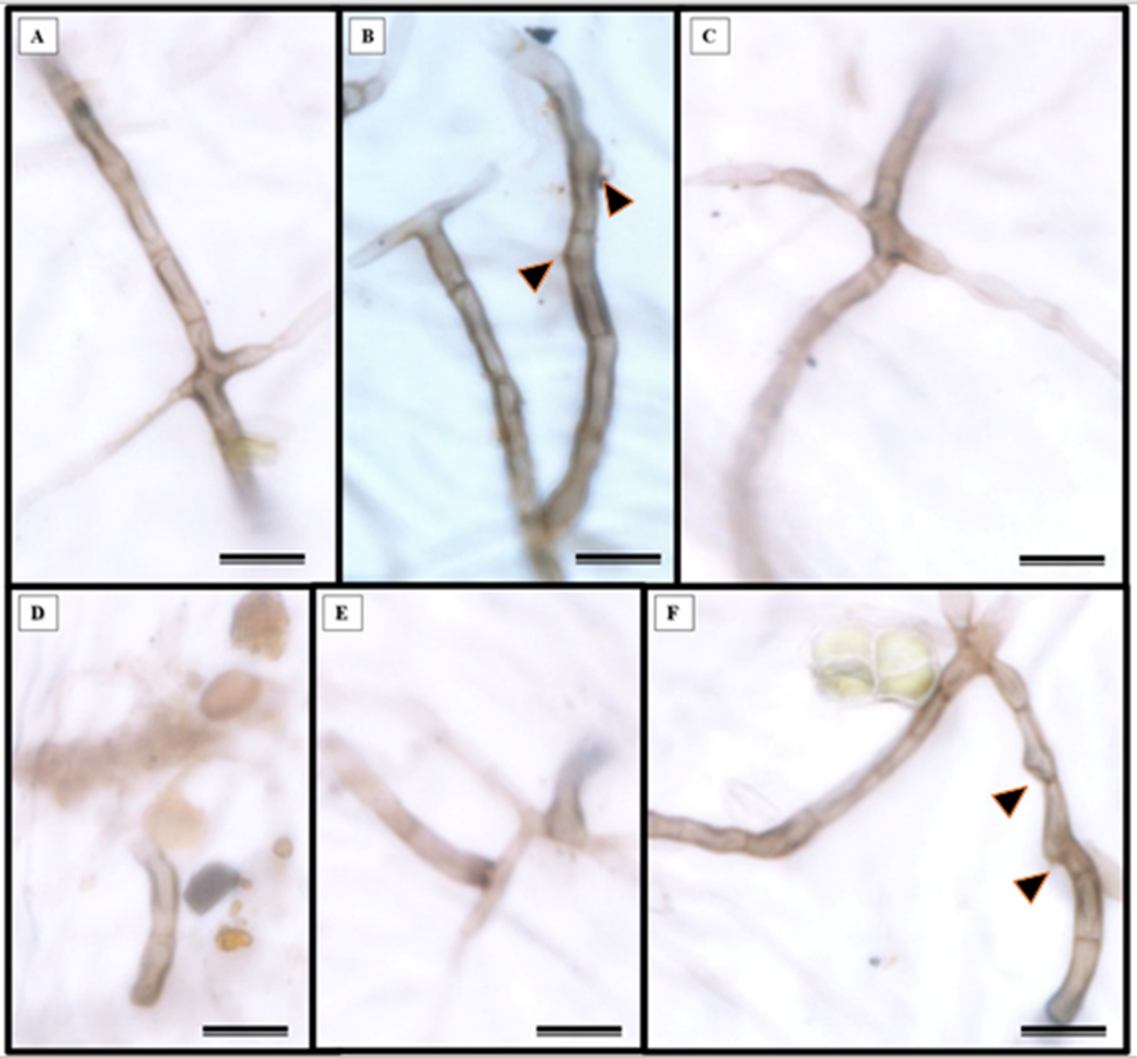
Presence of septate and melanized hyphae in the epidermis of the adaxial side of the *S. erecta* Radlk (Sapindaceae) leaf. (A–F) Arrows indicate the presence of conidiophores. 8 µm scale bar.

**Figure 3:**
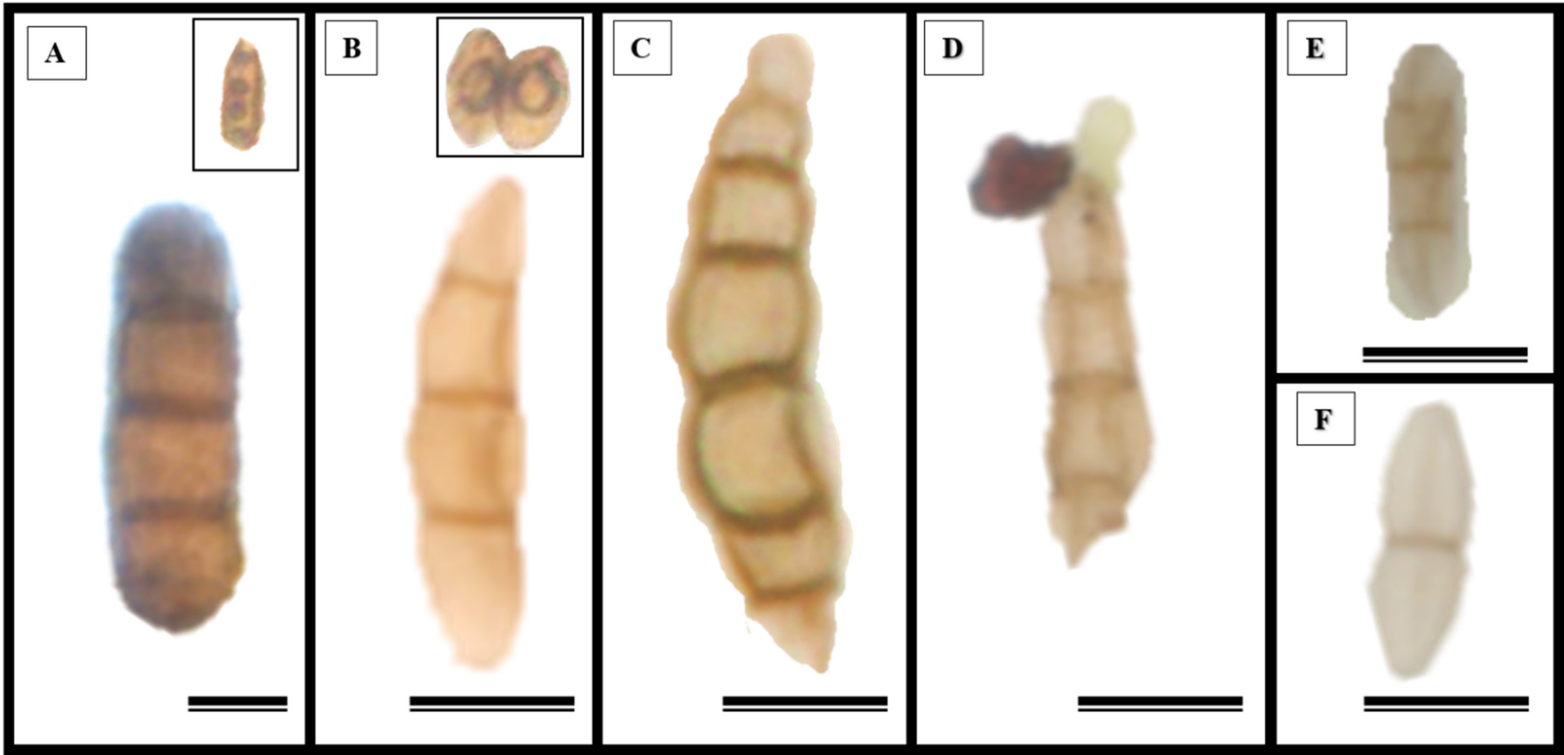
The presence of spores typical of *Bipolaris/Curvularia* anamorphs was observed in the epidermis of the *S. erecta* Radlk (Sapindaceae) adaxial leaf surface. (A–F) Conidia; (C and D) phragmospores; and (F) didymospores. 5.5 µm scale bar.

Epidermal impressions revealed the presence of septate, branched, melanized hyphae on the adaxial side of the *S. erecta* leaf samples (Figs. 2A–2F). These hyphae were identified as belonging to the *Bipolaris-Cochliobolus-Curvularia* complex because they have septate conidiophores, which are typically observed in this complex. *Bipolaris* and *Curvularia* are anamorphs of *Cochliobolus* in this complex. Colonization by the anamorphs was confirmed by the presence of typical spores, specifically conidia, that were subcylindrical to narrowly clavate and straight to slightly curved. Phragmospores and didymospores were also documented (Figs. 3A–3F).

**Figure 4:**
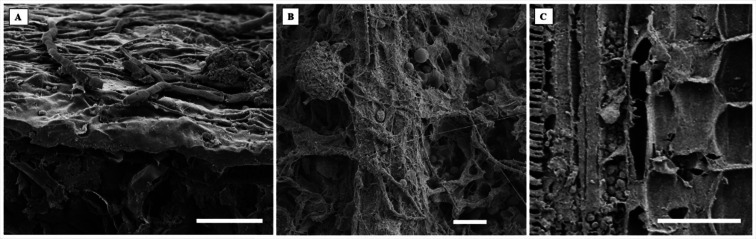
An electron micrograph of *S. erecta* Radlk (Sapindaceae). (A) Septate hyphae on the surface of the leaf epidermis; (B) hyphae and fungal spores within the inner tissues of the leaf lamina; and (C) microsclerotia in the phloem of the central leaf vein. (A) 100 µm scale bar; (B) 40 µm scale bar; and (C) 30 µm scale bar.

Electron microscopy images confirmed the presence of fungi that directly interacted with the adaxial epidermis of *S. erecta*. Septate hyphae were observed spreading across the tissue surface (Fig. 4A). On the contrary, hyphae and fungal spores were frequently observed colonizing the inner leaf tissues (Fig. 4B). Microsclerotia were observed in the phloem region (Fig. 4C).

### Leaf anatomical characterization

**Figure 5:**
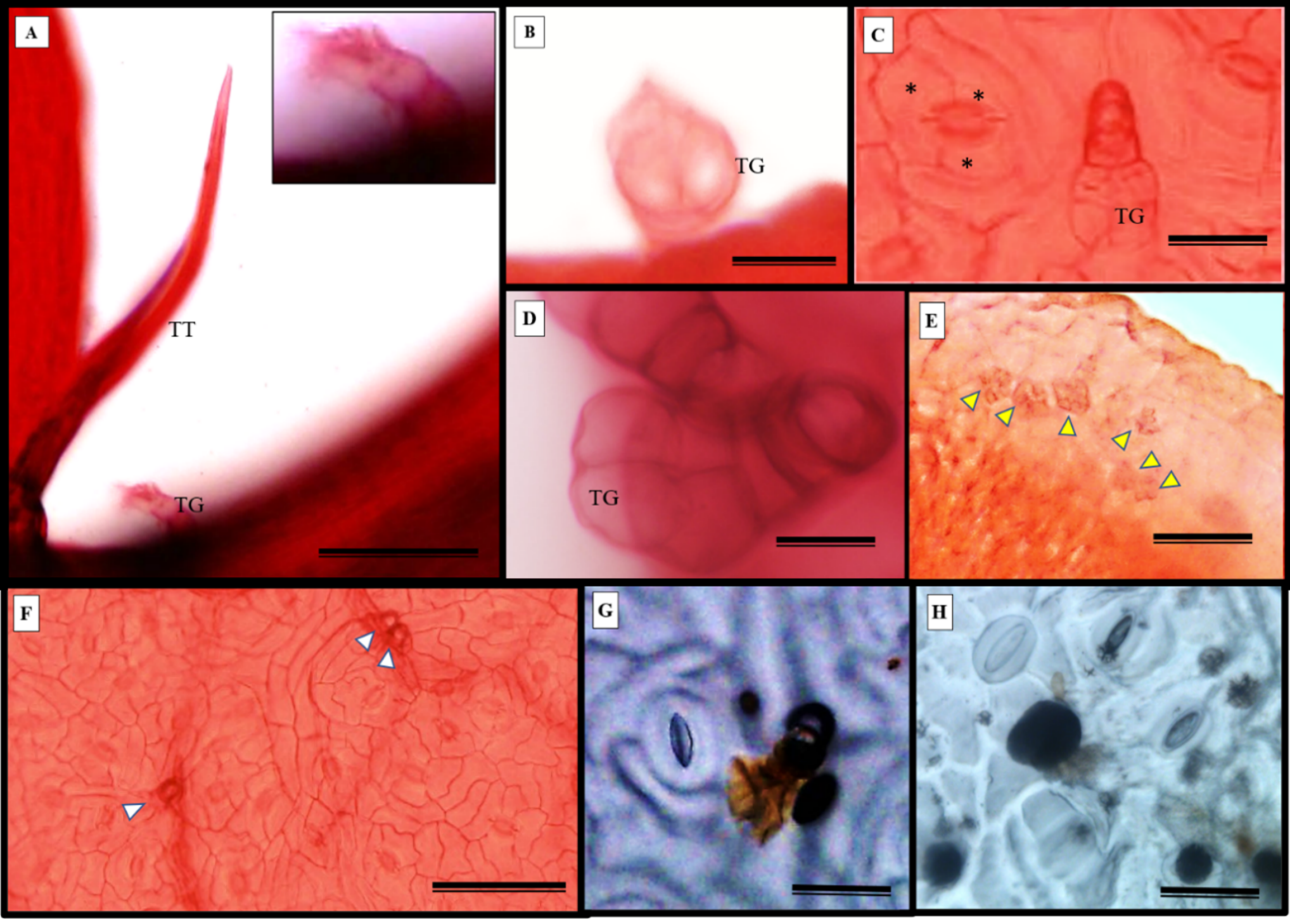
Fuchsin-stained diaphanization (A–F) and epidermal impression (G and (H) of *S. erecta* Radlk (Sapindaceae) under light microscopy. Apex of toothed leaf with tector trichome and glandular structures: (A) glandular trichomes; (B–D) stomata; (C, F–H) *S. erecta* toothed leaf with fungal microsclerotia; (E) tector trichome (TT), glandular trichome (TG); and an asterisk (*) indicates subsidiary cells. White arrows indicate glandular trichomes, and yellow arrows indicate microsclerotia. (A, D, and F) 100 µm scale bar; (B and H) 25 µm scale bar; (C and G) 50 µm scale bar; (E) 125 µm scale bar.

*S. erecta* leaves have both tectors and glandular trichomes, as shown in Figs. 5A–5B. The glandular trichomes are composed of a multicellular peduncle that is attached to a unicellular base. The multicellular head comprises two visible elliptical layers overlapping each other (Figs. 5B–5D). The observed stomata were classified as reniform and anisocytic (Figs. 5C, 5F–5H). Accumulations of melanized microsclerotia were observed in the leaf tooth region (Fig. 5E).

**Figure 6:**
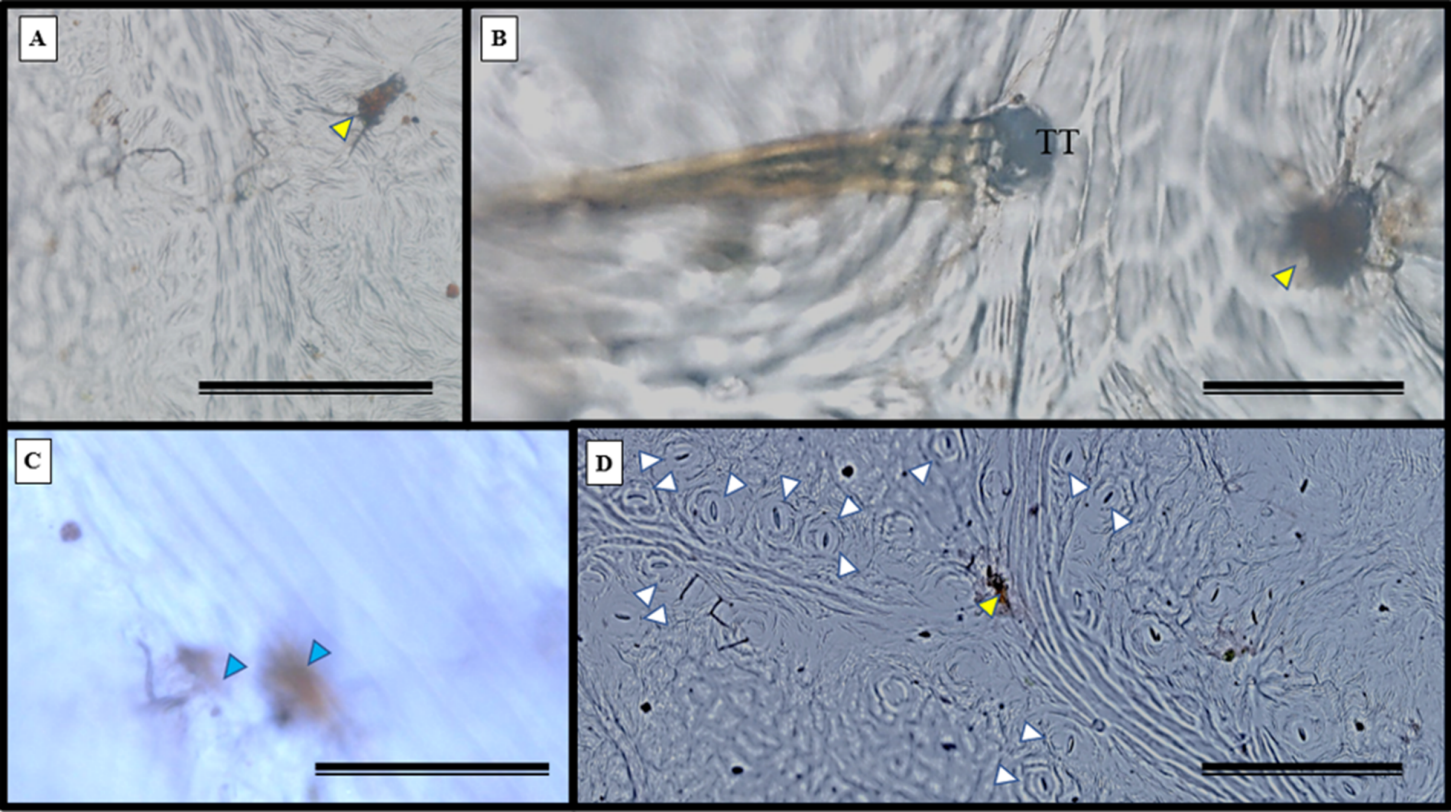
Impression of the *S. erecta* Radlk (Sapindaceae) epidermis in light microscopy. Adaxial side of *S. erecta*. (A) Glandular trichome; (B) tector and glandular trichome; (C) secretion from disruption of glandular trichome; (D) stomata and glandular trichome. Yellow arrows indicate glandular trichomes; blue arrows indicate secretion; and white arrows indicate stomata. TT, Tector Trichome. (A) 200 µm scale bar; (B) 50 µm scale bar; and (C–D) 100 µm scale bar.

**Figure 7:**
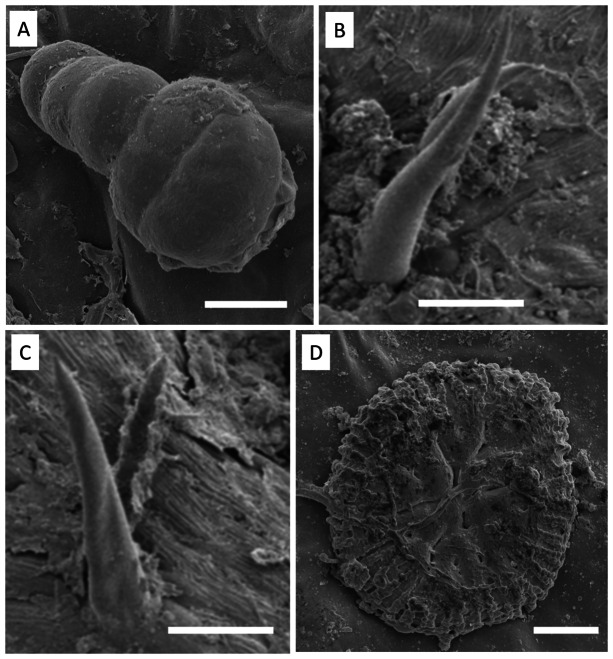
Electron micrographs of *S. erecta* Radlk (Sapindaceae) leaf teeth (A, B, and C) and leaf blade (D). (A) Glandular trichome; (B and C) tector trichomes; (D) scamiform trichome. (A and D) 10 µm scale bar; (B and C) 50 µm scale bar.

Glandular (Fig. 6A) and tectonic trichomes (Fig. 6B) were observed, as well as secretions resulting from rupturing due to mechanical pressure (Figs. 6B–6D). Electron microscopy images confirmed the presence of trichomes on the leaf teeth (Figs. 7A–7C) as well as scaly trichomes on the limbus (Fig. 7D).

**Figure 8:**
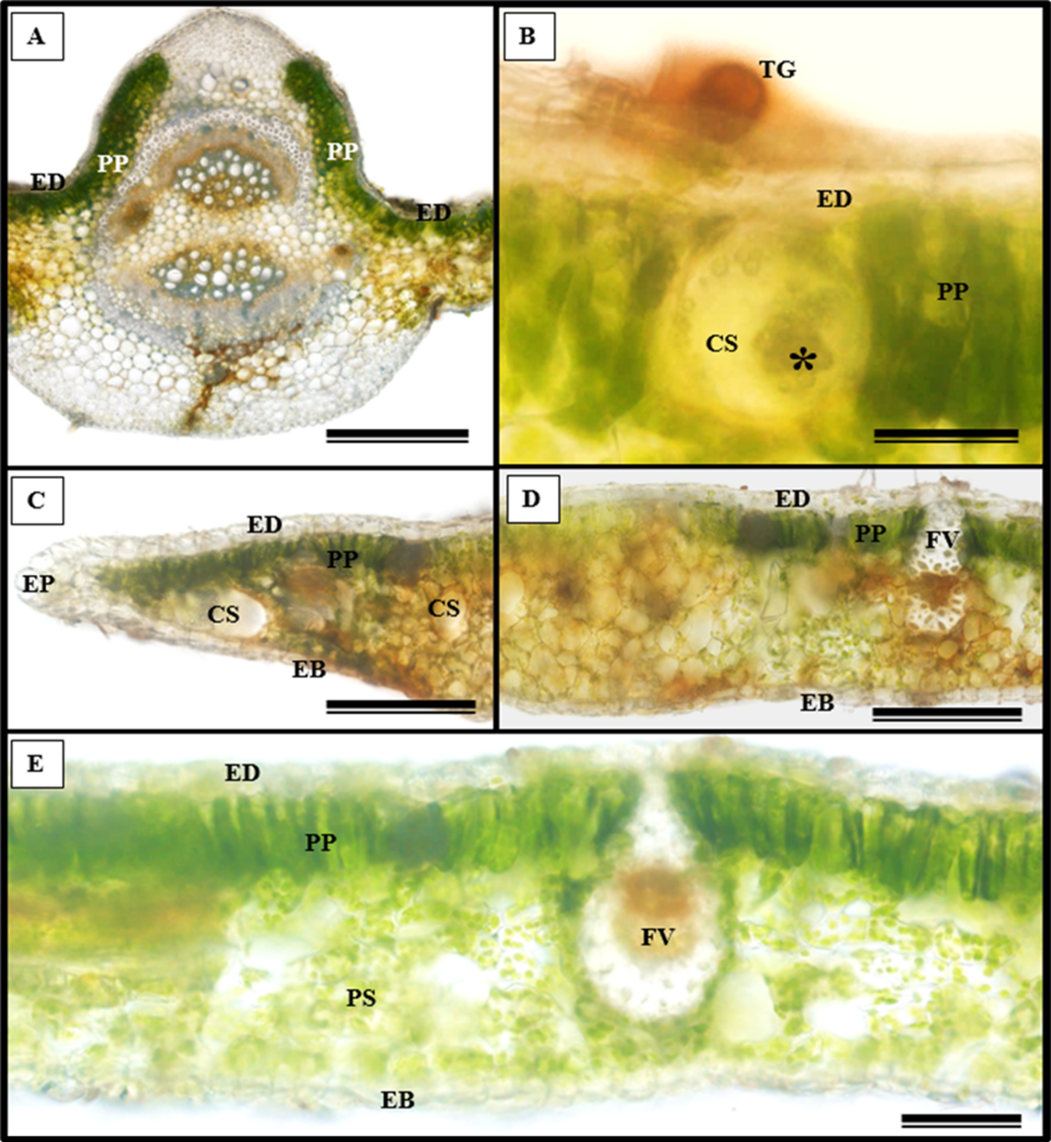
Transverse anatomical section of *Serjania erecta* Radlk (Sapindaceae) fresh leaves. (A) Biconvex primary vein; (B) secretory canal, fungus, and glandular trichome; (C) toothed leaf; (D) margin; (E) leaf limb. EB, Abaxial epidermis; PP, Palliçadic parenchyma; PS, Spongy parenchyma; CS, Secretory canal; ED, Adaxial epidermis; EP, Toothed leaf epidermis; VF, Vascular bundle; TG, Glandular trichome; an asterisk (*) indicates fungal microsclerotia. (A) 250 µm scale bar; B, 25 µm scale bar; (C–D) 100 µm scale bar; (E) 66 µm scale bar.

In addition to the teeth on the leaves, endophytic fungi were also observed colonizing the area surrounding the veins. *S. erecta* has a biconvex vein (Fig. 8A), and fungi can inhabit the secretory canal. Glandular trichomes were also observed on the adaxial epidermis, and exudates were present (Fig. 8B). Secretory channels were also observed in the vicinity of the toothed leaf (Fig. 8C). However, Fig. 8D indicates that the leaf margin does not possess any secretory structures. The species has mesophyll composed of a palisade and spongy parenchyma layer comprising four to eight cells (Fig. 8E).

**Figure 9:**
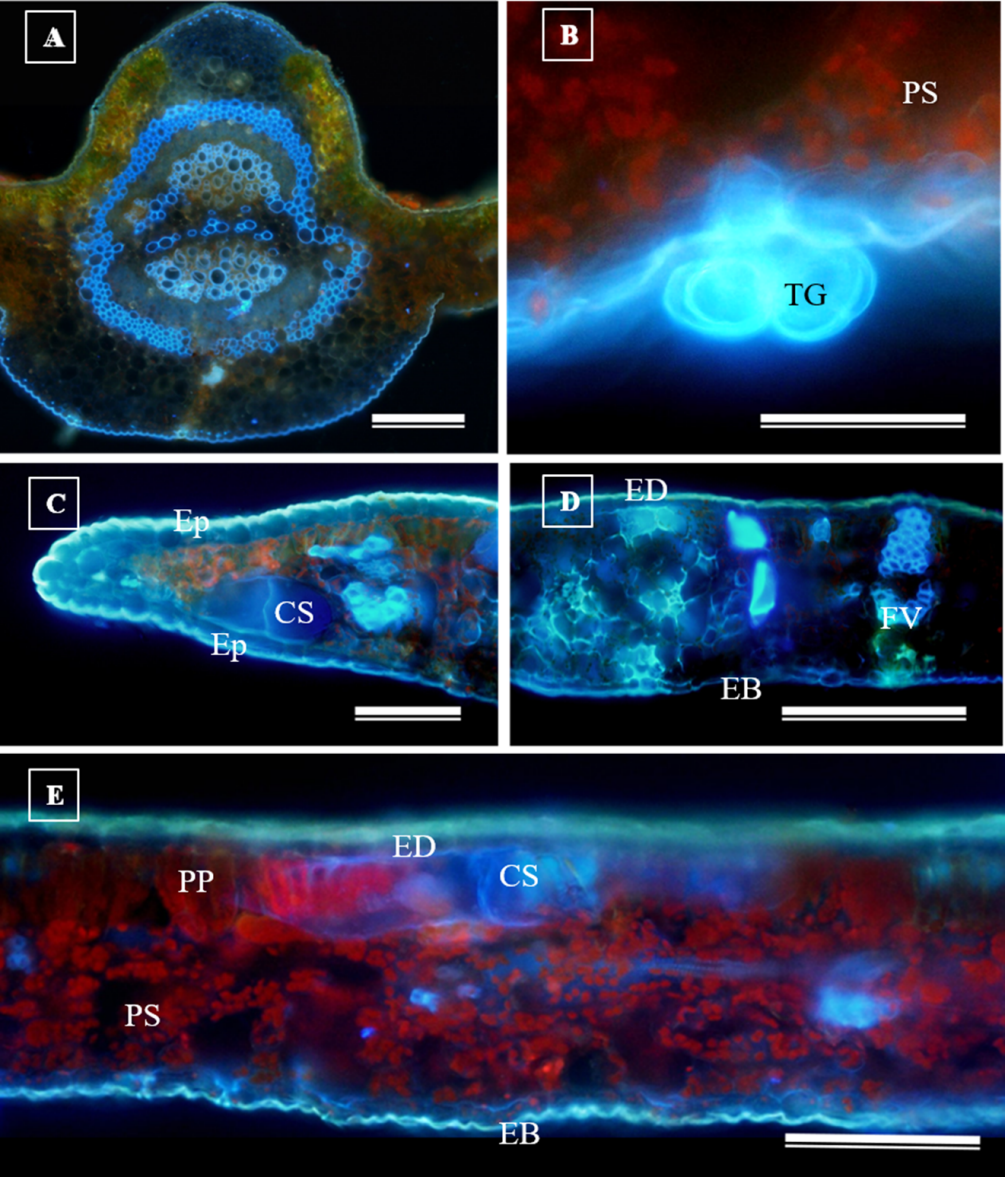
Transverse anatomical sections of fresh *S. erecta* Radlk (Sapindaceae) leaves were observed under ultraviolet light. (A) Biconvex primary vein; (B) glandular trichome; (C) toothed leaf; (D) margin; (E) leaf lamina. AE, Abaxial epidermis; PP, Palisade parenchyma; SD, Secretory duct; AE, Adaxial epidermis; Ep, Epidermis; VB, Vascular bundle; GT, Glandular trichome. (A) 250 µm scale bar; (B) 25 µm scale bar; (C–E) 100 µm scale bar.

Glandular trichomes are also present in the abaxial epidermis of *S. erecta* (Fig. 9). The secretory channel, located near the tooth and epidermal cells, emits blue fluorescence at approximately 460 nm (Fig. 9C). Fluorescence was also detected from the vascular bundles (Fig. 9D), vessel elements, and secretory channels in the epidermal cells (Fig. 9E).

### Histochemical tests

**Figure 10:**
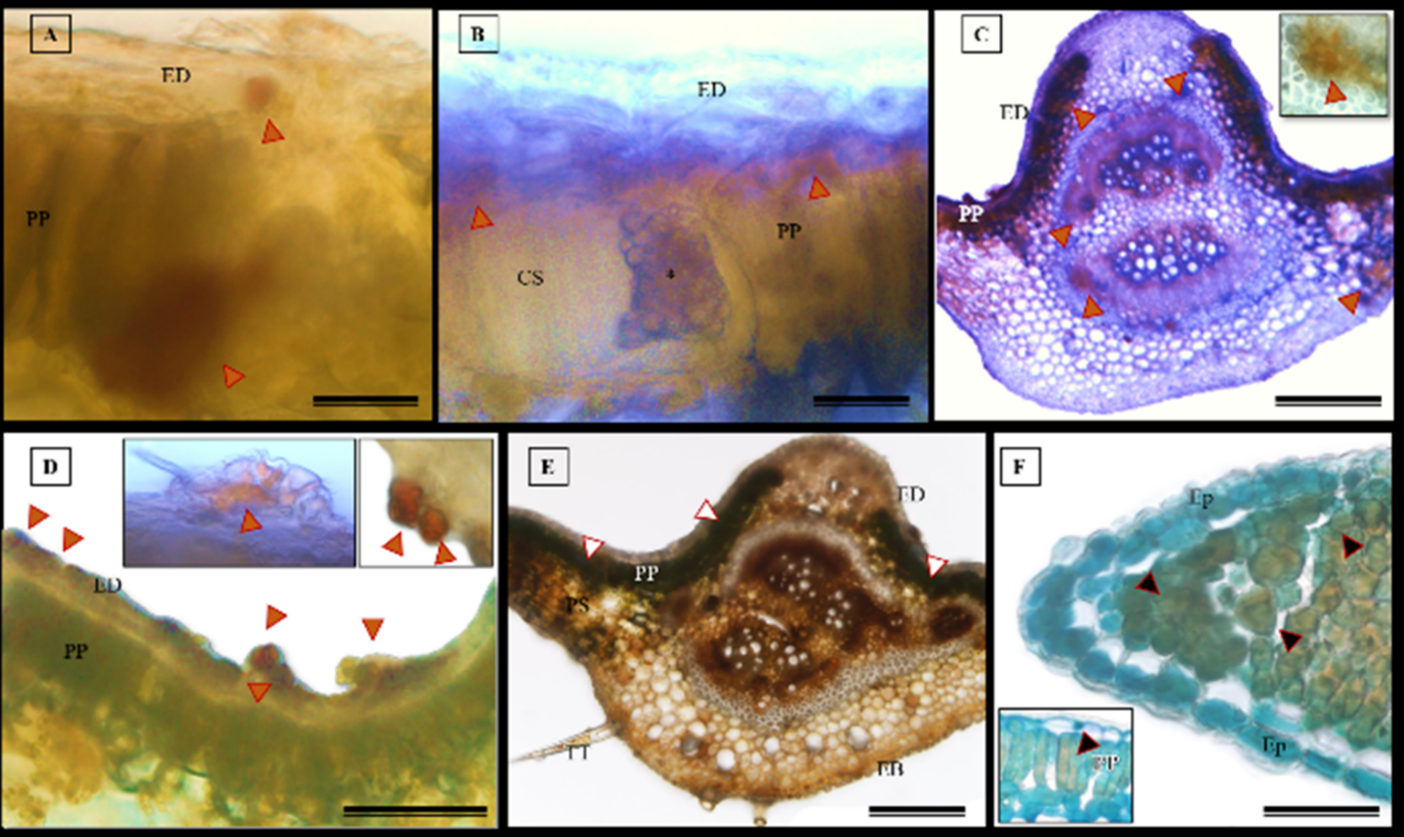
Histochemical tests were conducted to detect phenolic compounds in *S. erecta* Radlk (Sapindaceae) leaf specimens under light microscopy. (A–D) Hydrochloric vanillin; (E) potassium dichromate; (F) toluidine blue. ED, Adaxial epidermis; EB, Abaxial epidermis; PP, Palisade parenchyma; PS, Spongy parenchyma; CS, Secretory duct; TT, Tector trichome; an asterisk (*) indicates the presence of fungus. Arrows indicate the presence of phenolic compounds. (A and B) 25 µm scale bar; (C and F) 250 µm scale bar; (E) 100 µm scale bar.

The histochemical tests, namely hydrochloric vanillin, potassium dichromate, and toluidine blue staining, confirmed the presence of phenolic compounds in various tissues in *S. erecta* (Fig. 10), including the palisade parenchyma (Fig. 10A), the secretory canal amidst the fungal microsclerotia (Fig. 10B), the primary vein amidst the fibers and xylem walls (Fig. 10C), and the glandular structures in the epidermis (Fig. 10D). The potassium dichromate test detected phenolic compounds mainly in the palisade parenchyma and fibers found in the primary vein (Fig. 10E). Using the toluidine blue test, we were able to identify phenolic compounds in the cells of the palisade parenchyma and leaf teeth (Fig. 10F).

**Figure 11:**
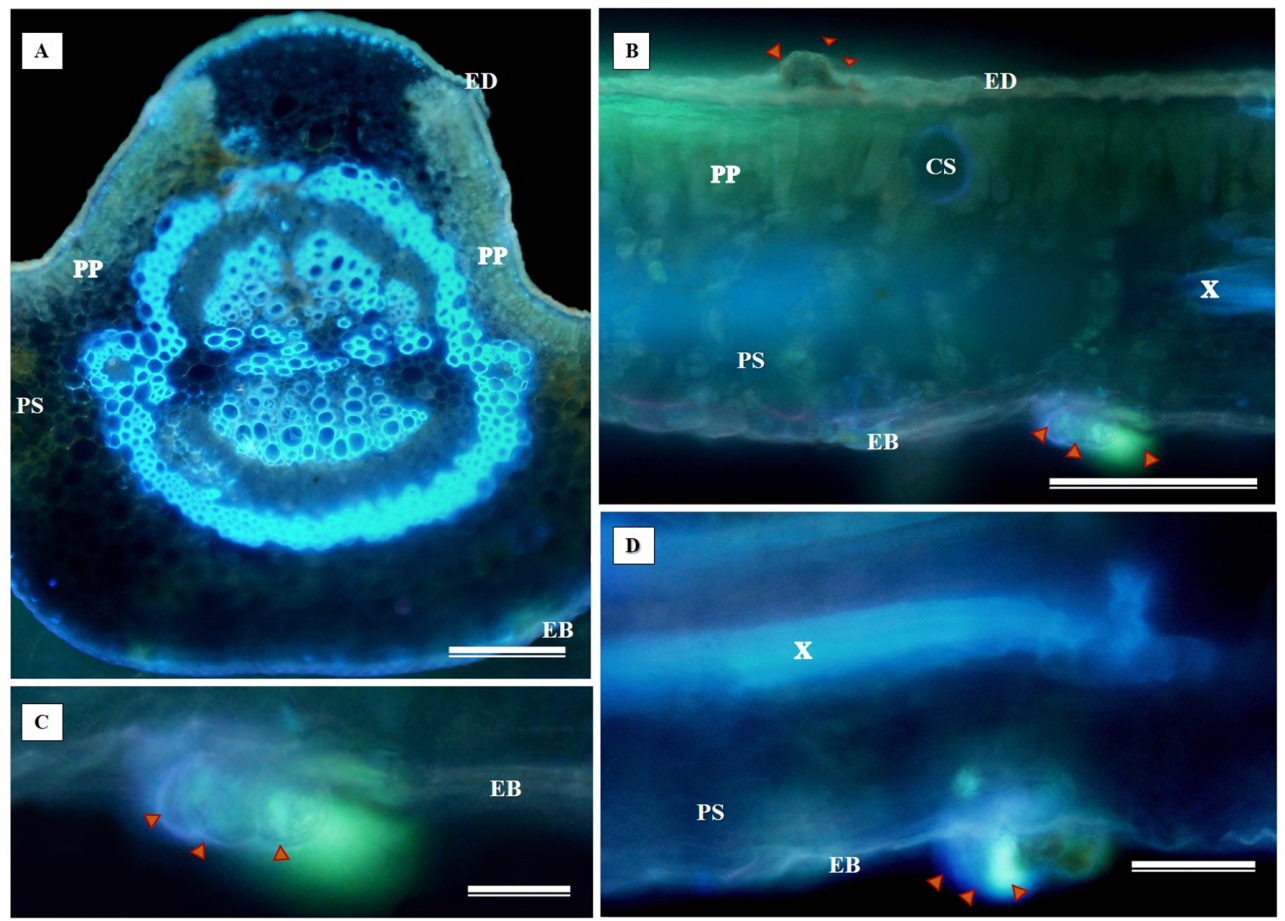
Histochemical testing for flavonoids using aluminum chloride was conducted on *S. erecta* Radlk (Sapindaceae) leaves. ED, Adaxial epidermis; EB, Abaxial epidermis; PP, Palisade parenchyma; SP, Spongy parenchyma; SD, Secretory duct; X, Xylem. Arrows indicate glandular secretory structures.

The histochemical test for flavonoids was performed using aluminum chloride, which stained the abaxial and adaxial epidermis as well as the cell walls of the vessel elements in the primary vein (Fig. 11A). The section xylem, abaxial epidermis (Fig. 11B), and glandular secretory structures (Figs. 11C–11D) were identified on the leaf limb.

**Figure 12:**
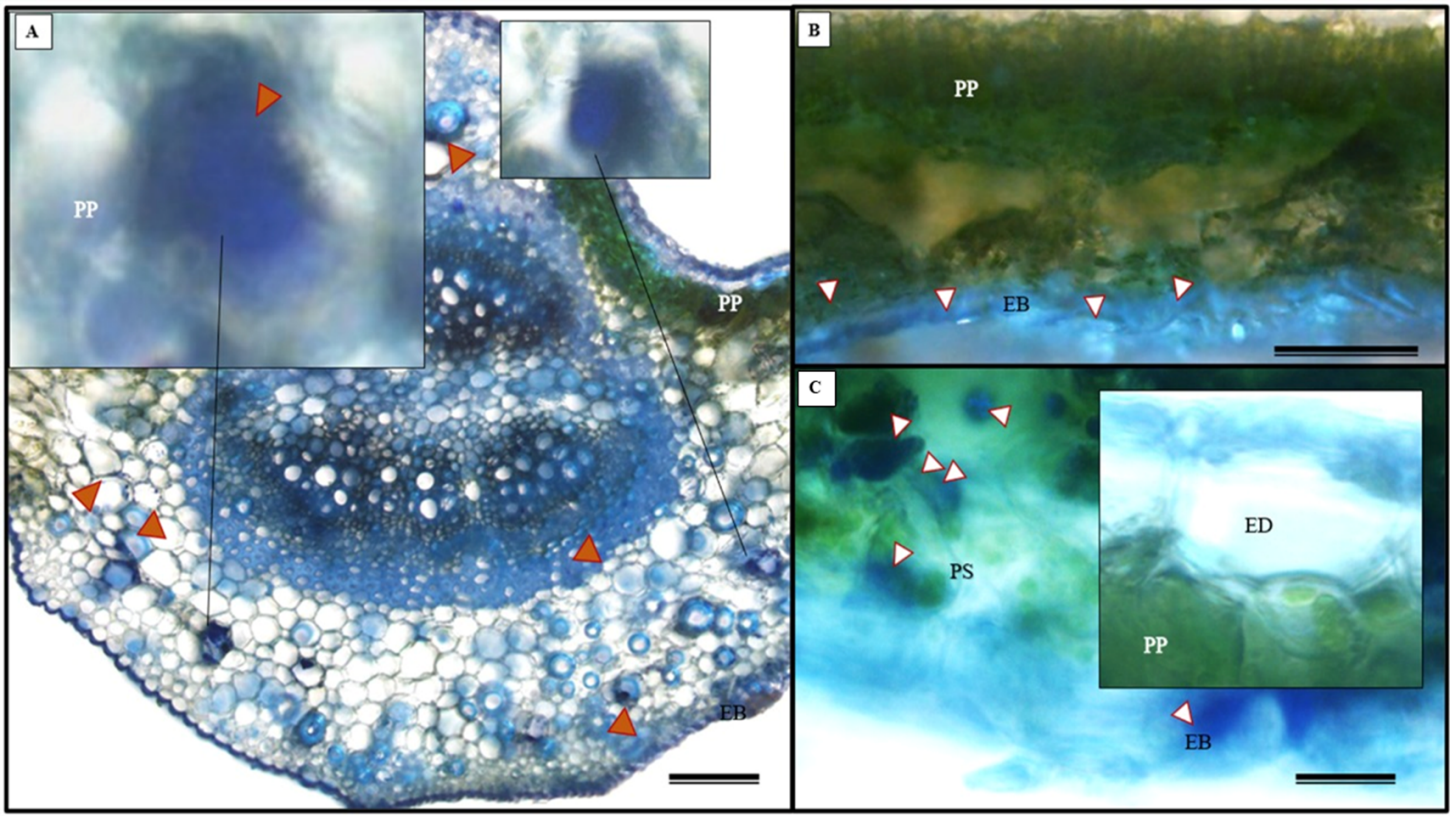
Histochemical test for essential oils in *Serjania erecta* Radlk (Sapindaceae) leaves under light microscopy. (A–C) NADI reagent. ED, Adaxial Epidermis; EB, Abaxial Epidermis; PP, Palisade Parenchyma; SP, Spongy Parenchyma. Arrows indicate the presence of essential oils. A and C represent 100 µm scale bars, while B represents a 200 µm scale bar.

NADI tests revealed that the adaxial epidermis tested positive for essential oils and oleoresins (Figs. 12A–12C).

**Figure 13:**
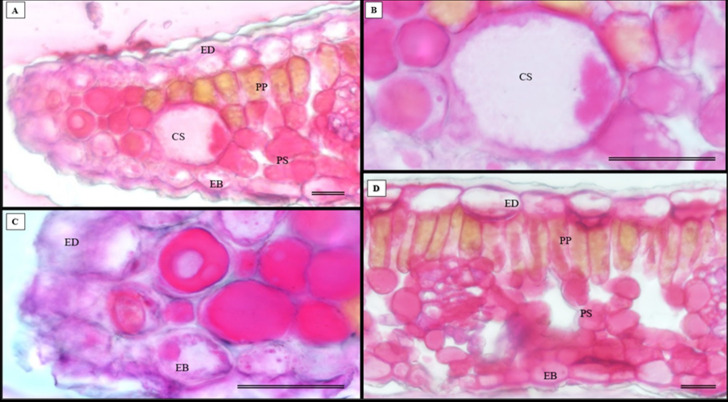
Histochemical testing for total polysaccharides (PAS) was conducted on *S. erecta* leaves under light microscopy. (A–D) Toothed leaf. ED, Adaxial Epidermis; EB, Abaxial Epidermis; PP, Palisade Parenchyma; PS, Spongy Parenchyma; CS, Secretory Duct. 25 µm scale bar.

The histochemical test for total polysaccharides using the periodic acid Schiff’s reaction (PAS) correctly identified the cell walls of *S. erecta* leaves (Fig. 13A), which comprise cellulose. Polysaccharides were marked within the secretory channel, which could indicate the presence of fungi (Fig. 13B). The presence of idioblasts, which contain polysaccharides, was observed in the leaf margins (Fig. 13C) as well as in the cells of the spongy parenchyma (Fig. 13D).

**Figure 14:**
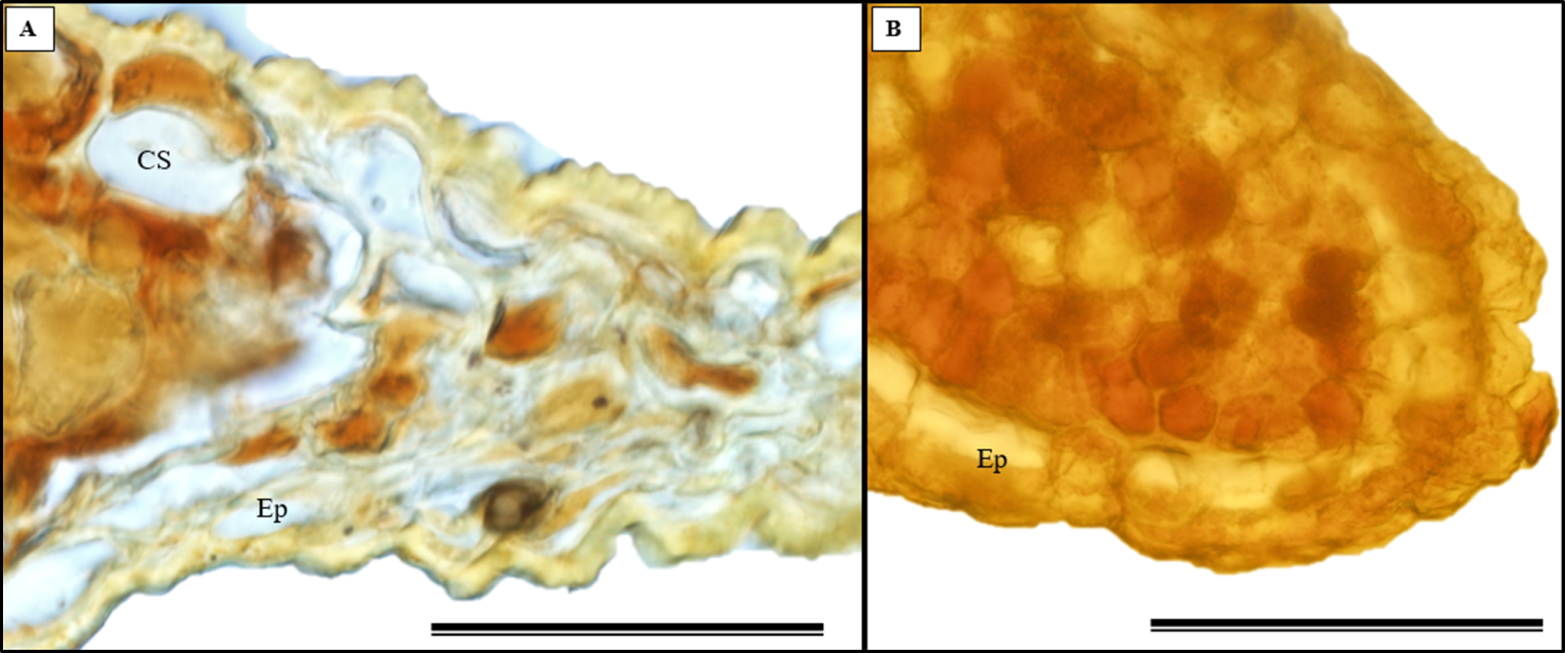
The histochemical test for alkaloids (using Wagner’s reagent) was conducted on *S. erecta* Radlk (Sapindaceae) leaves under light microscopy. Black indicates the presence of starch, while orange indicates the presence of alkaloid compounds. The bars represent 50 µm.

The histochemical test further confirmed the presence of alkaloids in the leaves, particularly in the leaf apices where idioblasts were observed (Figs. 14A and 14B).

## Discussion

### *Bipolaris* and *Curvularia* species colonize *S. erecta* leaf tissues

Many species of *Cochliobolus* have asexual states, which are also known as synonyms for both *Bipolaris* and *Curvularia*. Therefore, these fungi are classified within a species complex (Elkhateeb, Kolaibe & Daba, 2021). The presence of *Bipolaris/Curvularia* in the epidermis of *S. erecta* was evidenced by necrotic leaf spots on the limbus. These spots occur as a plant response to phytotoxin production by pathogens, such as ophiobolins synthesized by *Bipolaris* or radicinin produced by *Curvularia* (Srivastava et al., 2021; Xiao et al., 1991). Data suggests that phytotoxins synthesized by *Curvularia luneta* can cause general disruptions in the plasma membrane, leading to a decrease in active transport, which is reflected by the inhibition of protein precursors and loss of ions (Vianello, Macri & Passera, 1976). These toxins damage membranes, which trigger systemic responses in plants that ultimately result in cell death. *Bipolaris/Curvularia* fungi are phytopathogens of various plants, particularly grasses (Dos Santos et al., 2022; Gilardi et al., 2022; Ferdinandez et al., 2021). They are known to cause devastating epidemics globally in economically important food crops (such as wheat, rice, and corn) (Berbee, Pirseyedi & Hubbard, 1999; Scheffer, 1997). However, research shows that *Bipolaris/Curvularia* can establish themselves as endophytes in plants sampled from the Cerrado biome. Rosa et al. (2010) found that *Bipolaris/Cochliobolus* acted as endophytes in healthy *Piptadenia adiantoides* tissues*,* and Teles et al. (2005) isolated *Curvularia* sp. from *Ocotea corymbosa*, a plant native to this biome. This is the first report on the interaction between endophytic fungi and *S. erecta* tissues.

### Evidence of biotrophism has been observed in the interaction between *Bipolaris/Curvularia* and *S. erecta* leaf tissues

The first study to characterize leaf teeth, conducted by Hickey & Wolfe (1975), identified the teeth of the Sapindales order as Cunonioides (with a small glandular apex and the main vein running towards the apex). Within this order, the Sapindaceae family exhibits a wide range of indumentum variability, with non-glandular trichomes being more frequent. Multicellular glandular, papilliform, fasciculate, stellate, or scamiform trichomes occur less frequently, and stomata are classified as reniform and anisocytic (Tanaka, Pinto & Mourão, 2014; Acevedo-Rodríguez et al., 2011). The morphological data obtained for *S. erecta* support the observations made for Sapindaceae and indicate the presence of three types of trichomes (non-glandular, glandular, and scamiform) on the leaves of this species.

Trichomes are widely recognized as structural defense traits of plants (Da Silva & Batalha, 2011), which also serve as a barrier against water loss through evapotranspiration (Agrawal & Fishbein, 2006). Trichome density negatively affects the oviposition, feeding preference, survival, and growth rate of herbivorous insects (Agrawal & Fishbein, 2006). As a potential defense mechanism, *S. erecta* produces secretory structures near the toothed leaf. In this region, we observed the presence of tector, scabiform, and glandular trichomes, as well as secretions from the latter. In addition to the aforementioned protective strategies, *S. erecta* allows colonization of its epidermal surface by *Bipolaris/Curvularia*. This is evident from observing hyphae and conidia associated with epidermal cells. Furthermore, the plant provides colonization of its leaf tissues, as evidenced by melanized microsclerotia in intra-foliar structures such as secretory ducts and sieve tube elements. Research has demonstrated that phytopathogenic species can produce microsclerotia by establishing endophytic symbiotic relationships (Liu et al., 2022; Luo et al., 2019). Patil, Kulkarni & More (1966) showed that in *Curvularia* hyphae, aggregates of thickened or chlamydospore-like cells result in microsclerotia.

On the epidermal surface, fungi can act as a line of defense against pathogens (Wang, Wen & Asiegbu, 2022; Wen, Terhonen & Asiegbu, 2022) by exerting direct antibiosis. Benzopyrans from *Curvularia* exhibited antifungal activity against *Cladosporium sphaerospermum* and *Cladosporium cladosporioides* (Dos Reis, Do Vale & Lorenzi, 2022). Radicinin and its novel metabolite, O-demethylated-zeaenol, are effective in controlling the phytopathogens *Magnaporthe grisea* and *Valsa mali* (Yin et al., 2018; Zhang et al., 2011). Moreover, antifungal peptides have been isolated from pathogenic cultures of *Bipolaris* (Khiralla et al., 2019). A by-product mutualism interaction (Ruotsalainen et al., 2021) has been suggested between *Bipolaris/Curvularia* and *S. erecta*. This means that the investment made by *S. erecta* benefits *Bipolaris/Curvularia* as a by-product. In by-product mutualism, an organism benefits from the self-serving characteristics of another organism (Connor, 1995; Leimar & Hammerstein, 2010; Bronstein, 2015), as seen in microorganisms that utilize the metabolic waste products of their hosts (Harcombe et al., 2018). Evidence was found of endophytic fungi surviving in glandular regions and the phloem. In these regions, the primary and secondary metabolites of *S. erecta* are directed towards nourishing the hyphae and microsclerotia, which benefits the fungus.

Research has already demonstrated the saprophytic survival of *Bipolaris* and *Curvularia*, which depend on substrate degradation for nutrition (Stohr & Dighton, 2004; Duveiller et al., 2002; Reis et al., 1998). However, because of their saprotrophic ability to break down organic matter in soil and colonize plant tissues, fungi such as dark septate endophytes (DSE) could be classified as by-product mutualists. These fungi enhance the performance and vitality of their host plants by providing benefits without requiring significant investment from the host. Moreover, the literature has provided evidence of the transition from saprotrophic to hemibiotrophic and biotrophic states during evolution (Zhang et al., 2009). *Bipolaris/Curvularia* can be considered biotrophic when they derive their nutrition from the metabolism of *S. erecta*. Biotrophic pathogens can only survive by parasitizing living plant tissue. Thus, they rarely kill their hosts since they rely on them for nourishment. Evidence for a hemi-biotrophic lifestyle has already been proposed for *Bipolaris/Curvularia* (Lu et al., 2021; Poudel et al., 2019; Xue et al., 2008; Chen et al., 2003). Additionally, there are reports of a growth-promoting endophytic strain that, under the influence of a hormonal imbalance of indole acetic acid and brassinosteroids, can transform into a biotrophic pathogen (Yousaf et al., 2021).

### *Bipolaris/Curvularia*are insensitive to or feed on metabolites present in the toothed leaf of *S. erecta*

The removal of metabolites from the toothed leaf may be linked to the function of secretory channels and glandular trichomes. Some studies have linked these components to temperature gradients to understand their ecological and evolutionary importance (Peppe et al., 2011; Nicotra et al., 2011). Royer & Wilf (2006) hypothesized that toothed leaves increase sap flow, providing nutrients and other solutes to emerging and young leaves. In habitats with low temperatures, toothed leaves may be crucial in maximizing carbon gain potential and early-season growth. According to Hickey & Wolfe (1975), the presence of toothed leaves alongside glands is crucial to comprehending their function. The exudates observed from *S. erecta* trichomes may have a varied composition (Fahn, 1979) and are likely utilized in protection strategies against pathogen attacks or metabolized by biotrophic species.

### Histochemistry of *S. erecta*

Histochemical tests conducted on *S. pernambucensis*, another species of the genus *Serjania*, revealed that its stem contains a complex secretion consisting of various substances in three secretory structures: idioblasts, glandular trichomes, and laticifers (Da Cunha Neto et al., 2017). These data demonstrate the abundance of compounds detected in the leaves of *S. erecta*, including phenolic compounds, alkaloids, lipids, polysaccharides, proteins, essential oils, and other substances. In general, the presence of phenolic compounds is associated with responses to environmental stresses and/or plant protection (Jachula, Konarska & Denisow, 2018). Phytochemical analyses of plant extracts obtained from *S. erecta* have indicated the presence of phenolic compounds, including tannins and flavonoids (Gomig et al., 2008; Slomp et al., 2009; Broggini et al., 2010; Fernandes et al., 2011; Cardoso et al., 2013; Guimarães et al., 2015). These compounds protect the plant against herbivores and pathogens, attracting pollinators and dispersers. Furthermore, they can perform other functions, such as protecting against UV radiation or exhibiting allelopathic effects (Taiz & Zeiger, 2013). Studies have shown that these secondary metabolites can be produced directly by the plant or the endophytic fungi it hosts (Rocha et al., 2020; Amirzakariya & Shakeri, 2022).

Moreover, tannins isolated from *S. erecta* have already demonstrated inhibitory effects on *Bothrops jararacussu* venom (Fernandes et al., 2011). This suggests that the plant has anticoagulant properties and potential pharmaceutical applications. Guimarães et al. (2015) showed that *S. erecta* has three main flavonoids: quercetin, vitexin, and isovitexin. Vitexin protects PC12 cells (a cell line derived from an adrenal medulla pheochromocytoma) against toxicity. Additionally, it inhibits the induced generation of nitric oxide in these cells.

The detection of polysaccharides within the secretory cavity provides further evidence for the presence of fungi in these structures because fungi have cell walls composed of polysaccharides such as chitin and glycan (Curto et al., 2021; Brown, Esher & Alspaugh, 2020).

The presence of essential oils and oleoresins in *S. erecta* leaves was also observed. Resins of this type are composed of a combination of substances, including terpenoids, flavonoids, and lipids (Gupta et al., 2012). This study is the first to report the presence of essential oils and alkaloids in this species. Therefore, we recommend conducting additional research to clarify the composition of these oils and alkaloids and uncover their biotechnological potential. This study highlights the significance of conserving the biodiversity of the Cerrado region, not only for understanding how plants interact with microorganisms but also because these plants could potentially serve as sources for bioactive compounds in the future. This study aligns with initiatives to attribute value to the biodiversity of the Cerrado region and stimulate conservation initiatives and policies.

## Conclusions

*Bipolaris/Curvularia* species colonize *S. erecta* leaf tissues. Hyphae, conidiophores, and spores are observed in the adaxial epidermis, along with melanized microsclerotia in glandular regions and the phloem, which provide evidence of biotrophic behavior. The hypothesis that biotrophic phytopathogenic fungi interact with *S. erecta* leaf tissues was confirmed, despite large amounts of bioactive compounds as evidenced by histochemical analyses. On both the toothed leaf and epidermis of *S. erecta* tector, glandular, and scamiform trichomes have been demonstrated. Furthermore, we are reporting, for the first time, the synthesis of essential oils and alkaloids in the leaves of this species. Therefore, it is recommended that future studies focus on extracting and characterizing these compounds as well as exploring other aspects related to the interaction between *S. erecta* and the microorganisms in its microbiome.

## Additional Information and Declarations

### Field Study Permissions

The following information was supplied relating to field study approvals (i.e., approving body and any reference numbers): Field collections were allowed by the Post-Graduate Direction of the Instituto Federal Goiano Campus Rio Verde (project number: CMRV DPPG 011/2021).

### Data Availability

The following information was supplied regarding data availability: All data used in the preparation of the article are available in Figs. 1–14.
